# The Potential of Volatilomics as Female Fertilization Biomarkers in Assisted Reproductive Techniques

**DOI:** 10.3390/biomedicines14020264

**Published:** 2026-01-24

**Authors:** Ana Teresa Brinca, Maria Manuel Casteleiro Alves, Ana M. Peiró, Pilar Matallín Evangelio, Irene Eleno Buendicho, Antonio Helio Oliani, Vladimiro Silva, Ana Torgal, Luís F. Vicente, Ana Cristina Ramalhinho, Eugenia Gallardo

**Affiliations:** 1 RISE-Health, Medical Sciences Department, Faculty of Health Sciences, University of Beira Interior, 6200-506 Covilhã, Portugal; ana.brinca@ubi.pt; 2 Assisted Reproduction Laboratory, Cova da Beira Local Health Unit, 6200-251 Covilhã, Portugal; mariamanuelcasteleiro@gmail.com (M.M.C.A.); aholiani@gmail.com (A.H.O.); 3 Pharmacogenetic Unit, Clinical Pharmacology Department, Alicante Institute for Health and Biomedical Research (ISABIAL), Dr. Balmis General University Hospital, 03010 Alicante, Spain; peiro.anamaria@gmail.com; 4 Institute of Bioengineering, Miguel Hernández University, 03202 Elche, Spain; 5 Unidad de Reproducción, Hospital General Universitario de Alicante, 03010 Alicante, Spain; pmatallin@gmail.com; 6 Laboratorio de Fecundación In Vitro, Hospital General Universitario Dr. Balmis de Alicante, 03010 Alicante, Spain; ire_eleno@yahoo.com; 7 Gynaecology and Obstetrics, São José do Rio Preto School of Medicine, São José do Rio Preto 15090-000, Brazil; 8 Procriar-Centro de Procriação Medicamente Assistida, 4100-130 Porto, Portugal; vladsilva@ferticentro.pt; 9 Ferticentro-Centro De Estudos De Fertilidade S.A., 3000-316 Coimbra, Portugal; ana.torgal@ferticentro.pt; 10 Centro PMA, Hospital Lusíadas Lisboa, 1500-458 Lisboa, Portugal; luisferreiravicente@gmail.com; 11 Beiras Clinical Academic Centre (CACB), Group of Problems Related to Cancer, 6200-506 Covilhã, Portugal; 12 Laboratório de Fármaco-Toxicologia, UBIMedical, University of Beira Interior, 6200-284 Covilhã, Portugal; 13 Beiras Clinical Academic Centre (CACB), Group of Problems Related to Toxophilias, 6200-506 Covilhã, Portugal

**Keywords:** follicular fluid, volatile organic compounds, assisted reproductive technologies, fertilization-related parameters

## Abstract

**Background/Objectives:** Volatile organic compounds (VOCs) have emerged as promising non-invasive biomarkers for assessing metabolic and reproductive health. In the context of assisted reproductive techniques (ARTs), the volatilomic composition of follicular fluid (FF) may reflect the biochemical environment surrounding the oocyte, influencing fertilization success and embryo development. This study aimed to characterize the volatilomic profile of FF in women undergoing ARTs and to explore associations between specific VOCs and female fertilization-related parameters (FFRPs). **Methods:** A total of 54 Caucasian women aged 19–39 years, enrolled between October 2015 and July 2019, were recruited at the Assisted Reproduction Laboratory of the Local Health Unit of Cova da Beira, Covilhã. FF samples were analyzed via gas chromatography–mass spectrometry (GC–MS) in scan mode, identifying 136 VOCs, of which 72 were selected based on prevalence. Sixteen FFRPs were evaluated, including age, body mass index (BMI), smoking habits, infertility factor, oocyte yield, embryo quality, β-hCG levels, country of birth, and reproductive history. Associations between VOCs and FFRPs were assessed using the Chi-square (χ^2^) test. **Results:** Significant correlations (p ≤ 0.05) were identified between 45 VOCs and 11 FFRPs. The detected compounds comprised alkanes, siloxanes, aromatics, alcohols, ketones, aldehydes, carboxylic acids and esters, fatty acid derivatives, epoxides, acrylates, nitriles, and sterols. Several VOCs were associated with more than one FFRP, indicating overlapping metabolic pathways that may influence reproductive performance. **Conclusions:** The volatilomic profile of FF demonstrates significant variability linked to individual reproductive and metabolic factors. VOC analysis may provide novel insights into follicular physiology, representing a promising approach for identifying potential biomarkers of infertility and ART outcomes.

## 1. Introduction

Infertility, or subfertility, is recognized worldwide as a growing medical condition and social disorder, posing serious risks to contemporary reproductive health. It relates to both members of the couple [1], being described as the inability to conceive after 12 months of unprotected sexual intercourse [2] or due to impairment of the capacity to reproduce either as an individual or with a partner [3]. Around 15% of the world’s population is affected by this complex disease, with male and female factors contributing equally [4]. Approximately 85% of infertile couples have an identifiable cause, while the remaining 15% present with “unexplained infertility” [2,5]. Several factors adversely impact fertility rates both in men and women. Lifestyle and environmental factors and exposures, age [2], acute or chronic illnesses, infectious diseases, physiology, genetic abnormalities, and specific reproductive disorders make infertility highly individualized and challenging to address [1]. Due to social constructs, women tend to be the focus of assisted reproductive technologies (ARTs) [1,5]. Approximately 1 in 8 women, aged 15–49 years [2], seek medical attention and establish reproductive health evaluations at an earlier age [1]. The demand for infertility services has markedly risen, mainly due to trends of postponed childbirth and advancements in ATR [1]. ARTs have aroused much interest in recent years, but many variables influence the results of these procedures, rendering them challenging to optimize and accomplish [1,2,3]. Therefore, in addition to the currently available diagnostic and treatment tools, different approaches must be developed to improve the success of these procedures [1].

Follicular fluid (FF) plays a vital role in the bidirectional communication between oocytes and granulosa cells (GCs), regulating and promoting oocyte growth and development [6,7,8]. This biological matrix is the only one directly associated with the oocyte since it is where its growth and differentiation occur [9,10]. This fluid constitutes a complex microenvironment that derives from serum, transudate of plasma, and metabolites synthesized in the follicle wall that will later be altered by surrounding cells [6,9]. The FF compartment presents itself as a biomolecule-rich reservoir, in contrast to the dynamic nature of cells. The complex matrix is rich in hormones, growth factors, cytokines, lipids, proteins, and extracellular vesicles, which are essential for the oocyte’s growth [6,7,8]. Disruption of the intrafollicular environment under various clinical circumstances may affect the likelihood of becoming pregnant if therapy and/or corrections are not performed promptly [10,11]. FF is a non-invasive matrix for fertility insights, reflecting changes in patients’ microenvironments. Understanding FF and its composition is crucial for investigating pathologies and prognosis of diseases. Given its interactions with the oocyte, FF may be able to forecast the quality of the oocyte, providing vital information regarding fertilization techniques and the likelihood of fertilization and embryo implantation [6].

Volatile organic compounds (VOCs) comprise an exciting part of the metabolome [12]. They are small (<500 Da) [13] thermostable molecules directly linked to multiple metabolic and biochemical processes, making them a target for investigations in stand-alone or associated with other approaches [1,12]. Given their properties, they can be easily sampled from readily accessible biofluids, paving the way for a non-invasive, straightforward, fast, risk-free, affordable and simple detection approach compared to other technologies, which can be more favourable for wide application [1,12]. Additionally, it requires little or no sample preparation, rendering volatilomics as a potentially green approach in analytical chemistry [13]. Pathophysiological alterations associated with disease onset and progression can modulate the volatilomic profile, enabling the delineation of diagnostic biosignatures. These volatilomic shifts reflect deviations from homeostasis, support the characterization of disease trajectories, and facilitate the evaluation of therapeutic responses, thereby aiding in the identification of clinically relevant biomarkers [1,13,14]. Furthermore, volatilomics studies can range from targeted analysis of one or a small number of metabolites associated with a specific biological pathway, to the fingerprinting of a large subset of metabolites associated with a particular phenotype or stimulus. Untargeted approaches are more appropriate to detect unexpected changes in the concentrations of specific metabolites [13]. Volatilomic-based tests can be applied across diverse clinical conditions and offer a promising approach to overcome current diagnostic challenges. Moreover, they hold substantial value for distinguishing clinical phenotypes within patient groups, thereby supporting more personalized therapeutic strategies [12,13,14].

Despite growing interest in metabolomics and lipidomics in reproductive medicine, few studies have applied volatilomics to assess female reproductive potential. Previous reports have mostly focused on serum or urine matrices, leaving FF relatively unexplored. Thus, studying the volatilomic composition of FF offers a unique opportunity to capture the biochemical environment directly surrounding the oocyte, potentially identifying non-invasive biomarkers predictive of ART success.

The aim of the present research was to examine the relation between factors associated with female fertilization procedures and the volatilomic profile of the FF from women who underwent ART. The study hypothesis is that specific specimens of VOCs might be associated with these parameters and, therefore, influence the efficacy of the procedures in question.

## 2. Materials and Methods

### 2.1. Subject Sample Collection

To find potential biomarkers that relate to successful fertilization procedures, the volatomic pattern of the FF from 54 individuals who underwent ARTs was examined. The collection of FF and related data followed the protocol established in a previous study [15]. Women were enrolled between October 2015 and July 2019. All subjects were Caucasian, with age ranging from 19 to 39 years old. The samples were obtained at the Assisted Reproduction Laboratory of the Local Health Unit of Cova da Beira in Covilhã, Portugal. These included patients with polycystic ovary syndrome (PCOS), endometriosis, premature ovarian failure (POF), and controls. The controls corresponded to women subjected to *in vitro fertilization* (IVF) procedures due to specific conditions that did not affect the FF, such as tubal obstruction, or when the couple’s primordial fertility factor was male-driven. Some patients presented more than one clinical condition.

### 2.2. FF Sample Collection and Preparation

FF sample collection, preparation, and VOCs extraction were performed following the same analytical procedures as in our earlier studies [1,15]. Oocyte retrieval was performed by transvaginal ultrasound-guided aspiration 36 h after the injection of human chorionic gonadotrophin, and each follicle was aspirated. Blood contamination was avoided, and only clear fluid samples were included. All FF samples from the same patient were pooled, and a volume of 15 mL was centrifuged at 3000× *g* for 15 min. All samples were processed immediately upon availability. As soon as each sample arrived from the hospital, it was filtered and stored at −80 °C following a standardized procedure, always performed by the same trained operator. Supernatants were filtered with 0.2 um filters to eliminate cell debris and then stored at 80 °C.

### 2.3. VOCs Extraction and Analysis

Samples were stored at 4 °C prior to analysis. For each FF sample, 2 mL was placed in a vial, and volatile metabolites were extracted using a 100 µm polydimethylsiloxane (PDMS)-coated solid-phase microextraction (SPME) fiber (Supelco, Bellefonte, PA, USA) exposed in the vial headspace for 45 min at 40 °C under continuous agitation (125 rpm). Fibers were conditioned according to the manufacturer’s instructions, and VOCs were desorbed in the GC injection port for 5 min. PDMS fibers were chosen for their suitability for analytes with molecular weights of 80–500 Da and compatibility with a manual holder. To monitor potential environmental or procedural contamination, a blank control was included, consisting of an empty vial exposed to the same SPME fiber under identical conditions. The volatilomic profiling of all samples was carried out in a single batch in 2022 by the same operator; therefore, no inter-batch variation was introduced in this study.

Headspace VOCs were analyzed using an HP 7890B gas chromatograph coupled to an Agilent 5977A mass spectrometer and Agilent 7693 autosampler. Separation was performed on a 30 m × 0.25 mm I.D., 0.25 µm HP-5MS capillary column (J & W Scientific, Folsom, CA, USA). The oven program was 45 °C for 5 min, ramp to 150 °C at 2 °C/min, hold 10 min, ramp to 220 °C at 7 °C/min, and hold 10 min. Helium was used as the carrier gas at 1.0 mL/min. The injection port was maintained at 250 °C in splitless mode for 5 min.

The mass spectrometry parameters were transfer line 280 °C, quadrupole 150 °C, ionization source 230 °C, electron impact 70 eV, ionization current 35 µA, scan range *m*/*z* 50–550. VOCs were identified using Agilent MS ChemStation (version B.04.03) with NIST20, Wiley12, and SWGDRUGv8 libraries (similarity >80%) or commercial standards when available.

The complete experimental procedure has been thoroughly detailed in our previous studies, where all methodological steps, analytical conditions, and quality-control measures are fully described [1,15].

### 2.4. Study Variables

The variables in the study encompassed 16 female fertilization-related parameters (FFRPs). These included age, body mass index (BMI), smoking habits, infertility factor and time trying to conceive, number of aspirated oocytes aspirated, the total dosage of gonadotropins administrated, the quality of the embryos transferred, the levels of β-hCG administrated, total embryo quality, country of birth, and the number of previous pregnancies. Table 1 shows the different data regarding FFRPs and their specific descriptions. These were correlated with the volatilomic profile of each FF sample. Overall, 136 VOCs were determined via scan mode in all 54 FF specimens, with 72 compounds being studied.

### 2.5. Data Analysis

To evaluate whether the available sample size (N = 54) provided adequate sensitivity to detect associations, we estimated the median observed effect size for each clinical parameter by computing Spearman correlations across all 72 volatile organic compounds (VOCs). Spearman’s method was chosen because VOC data are typically non-normal, highly variable, and often subject to detection limits, making rank-based correlations more robust in this context. The median correlation was used instead of the mean to obtain a stable estimate of the typical effect size in this highly dimensional dataset.

A post hoc power analysis was then conducted in G*Power (3.1.9.7) using these median observed correlations as the effect size (α = 0.05, two-tailed). The resulting statistical power (1–β) ranged from 0.0700 to 0.1826, indicating that the sample size of 54 provides limited sensitivity for detecting small effects. This is consistent with expectations for VOC datasets, where true correlations tend to be modest. Larger samples would be needed to reach conventional levels of statistical power (e.g., 0.80).

It should be noted that the study population includes individuals with different infertility conditions, namely, endometriosis, PCOS, POF, and controls. These clinical entities are known to influence follicular physiology and could therefore act as potential confounding factors. However, due to the limited sample size and the large number of VOCs and clinical parameters analyzed simultaneously, stratification by infertility diagnosis was not feasible in the present study without severely compromising statistical power and interpretability. Importantly, the impact of these specific infertility conditions on the follicular fluid volatilomic profile has been partially addressed in a previously published study by our group, in which volatilomic differences between endometriosis, PCOS, POF, and control patients were specifically investigated [1]. As such, the present work was deliberately designed to focus on associations between VOCs and general female fertilization-related parameters, rather than on diagnostic group comparisons.

Multivariate statistical analysis resorting to Chi-square test (χ^2^) were conducted to assess the association between the VOCs and FFRP, regarding *p*-value as ≤0.05. For variables with low cell counts in some categories (e.g., country of birth), Fisher’s exact test was performed instead of the Chi-square test to ensure the validity of the analysis (IBM SPSS Statistics (version 29)).

Spearman correlations were calculated between individual VOCs and clinical parameters to assess potential associations. Due to the large number of comparisons, *p*-values were adjusted for multiple testing using the Benjamini–Hochberg false discovery rate (FDR) procedure. Correlations with q < 0.05 were considered statistically significant (IBM SPSS Statistics (version 29)). This approach was chosen because VOC data are sparse and highly variable, with many compounds appearing in only a few samples. Therefore, tests such as multivariate regression were not considered appropriate for the main analyses, as they would be unreliable given the sample size and distribution of the data.

## 3. Results

A total of 136 VOCs were determined via scan mode in all 54 specimens of FF. Due to their prevalence throughout the samples, only 72 compounds were studied. The considered metabolites were present in at least two specimens. The chosen VOCs were present in at least two of the overall samples. A total of 16 FFRP were analyzed. Multivariate statistical analysis yielded significant alterations and correlations between 45 of the VOCs and 11 FFRP. As presented in Table 2, the FFRP encompass age, BMI, smoking habits, infertility factor, total dosage of gonadotropins given to the patients, aspirated oocytes, total embryo quality, β-hCG, country of birth, and nº of previous pregnancies. The VOCs include alkanes (straight-chain and branched saturated hydrocarbons), siloxanes (cyclic silicon–oxygen compounds), aromatic hydrocarbons (benzene derivatives), alcohols, ketones, aldehydes, carboxylic acids and derivatives, fatty acid derivatives, epoxides, acrylates and nitriles (reactive groups), sterols (biologically important lipids), and other esters (specialized compounds for fragrances, coatings, etc.).

After applying FDR correction, only the correlation corresponding to the lowest *p*-value remained statistically significant (q = 0.044). All other correlations did not reach statistical significance after adjustment (q > 0.05). These results indicate that, while some associations appeared in uncorrected analyses, the majority of compound–clinical parameter relationships are weak or limited by the sparse distribution of VOCs and the sample size. Accordingly, Chi-square tests of association provided a useful exploratory overview of presence/absence patterns, even though few associations reached statistical significance.

### 3.1. Age

Two upper-threshold categories of age showed relevance according to the analytes in study, namely Age 35 and Age 37. Cyclotetradecane was detected in 5 of the 29 women aged between 35 and 39 years, while undecane-4,8-dimethyl, decane-2,4,6-trimethyl, and tetradecane were each found in 2 of the 19 women aged between 37 and 39 years, suggesting that their presence may be linked to older reproductive age. These findings suggest potential age-associated shifts in the follicular volatilomic profile, possibly reflecting metabolic and oxidative changes accompanying reproductive aging. Table 3 summarizes the presence of selected VOCs identified in women aged ≥35 and ≥37 years undergoing assisted reproduction. Data are presented as the number of positive samples and corresponding percentage relative to the total cohort (n = 54).

### 3.2. Body Mass Index

Tetradecamethylcycloheptasiloxane was observed in 5 of the 13 women with BMI above 25 and in 30 of the 41 women with BMI at or below 25, meaning 64.8% of all samples contained this compound, of which 85.7% were in women with BMI ≤ 25, indicating a strong association with lower BMI. Benzene-2-ethyl-1,4-dimethyl appeared in 2 of the 13 women with BMI > 25, representing 3.7% of all samples, with 15.38% of the women showing it belonging to the higher BMI group. Tetramethylbenzene was found in 3 of the 13 women with BMI above 25 and in only 1 of the 41 with BMI ≤ 25, so that 5.55% of all samples presented this compound and 75% of the women with it were overweight. Eicosamethylcyclodecasiloxane and octadecan-1-ol trimethylsilyl ether appeared in 3 of the 13 women with BMI above 25 and 2 of the 41 with BMI ≤ 25, with 5.55% of all samples showing them and 60% belonging to the higher BMI category. These variations may reflect BMI-associated metabolic or oxidative alterations within the follicular environment, potentially influencing oocyte microchemistry and reproductive outcomes. Table 4 summarizes the detection frequency and percentage of relevant VOCs grouped by BMI ≤ 25 and >25.

### 3.3. Smoking Habits

Smoking habits revealed further distinctions. 2-Nonadecanone and tetratriacontane were found in 1 of the 6 women with past smoking habits, while stearyl alcohol was detected in 4 of the 43 never-smokers, 3 of the 6 past smokers, and 1 of the 5 current smokers. Notably, stearyl alcohol was the most prevalent compound among previous smokers, while the appearance of long-chain hydrocarbons and fatty alcohols suggests possible smoking-related metabolic or lipid peroxidation effects within the follicular microenvironment. Table 5 presents the detection frequency and percentage of selected VOCs regarding smoking habits, categorized as never, previous, or active smokers.

### 3.4. Infertility Cause

Infertility status showed even stronger patterns: hexadecanoic acid trimethylsilylester was present in 9 of the 39 women who were considered the primary cause of infertility of the couple, corresponding to 23.08% of that subgroup and 16.67% of all samples, whereas hexadecanal was found in 6 of the 15 controls and 3 of the 39 infertile women, and oxirane-hexadecyl appeared in 5 of the 15 controls and 3 of the 39 infertile women. Cyclopentasiloxane-decamethyl and decane were detected in 2 of the 15 controls, corresponding to 3.70% of all samples and 15.38% of all controls. Aldehydes and siloxanes were more frequently detected in women without an infertility factor, whereas long-chain fatty acid derivatives were prevalent in those with an identified infertility factor, suggesting possible differences in lipid metabolism or FF composition related to reproductive health status. Table 6 summarizes the detection frequency and relative proportion of relevant VOCs present in the FF of women undergoing assisted reproduction, categorized by presence or absence of a diagnosed infertility factor.

### 3.5. Gonadotropin Dosage

With respect to the total dosage of gonadotropins received during ovarian stimulation, palmitic acid was present in 3 of the 35 women who received less than 3000 IU and in 8 of the 19 who received 3000 IU or more. Phenol and isopropyl myristate were present in 1 of the 35 low-dose women and 4 of the 19 high-dose women, while palmitic acid methyl ester was detected in two of the low-dose groups and five of the high-dose groups. Stearyl alcohol appeared in two of the low-dose groups and six of the high-dose groups. Octadecanoic acid and cyclotetradecane were both detected in 5 of the 19 high-dose women, representing 9.26% of all samples and 29.32% of the high-dose subgroup, while octadecanoic acid methyl ester was found in four of these women, corresponding to 7.41% of all samples and 21.05% of the high-dose subgroup. 1-Dodecanol and oleamide were both present in three of the high-dose groups, or 5.56% of all samples and 15.79% of that subgroup. Trimethylsiloxyhexadecane, pentadecane, octane-1,1′-oxybis, 2-ethylhexyl salicylate, nonadecane, 2-propenoic acid-3-(4-methoxyphenyl)-2-ethylhexyl, and dihydro-methyl-jasmonate were found in 2 of the 19 high-dose cases, accounting for 3.70% of all samples and 10.53% of the high-dose subgroup. Women receiving higher gonadotropin dosages (≥3000 IU) showed a broader range and higher frequency of lipid-derived and aromatic VOCs, suggesting possible dose-dependent alterations in FF lipid metabolism and oxidative processes during controlled ovarian stimulation. Table 7 details the occurrence and relative frequency of VOCs stratified by total gonadotropin dosage received (<3000 IU vs. ≥3000 IU).

### 3.6. Number of Aspirated Oocytes

Ovarian response was analyzed via the number of aspirated oocytes collected through follicular aspiration. Two stratified groups were considered, however, only the parameter related to ≤10 and >10 aspirated oocytes revealed significant relevance, with the parameter concerning ≤6 and >6 aspirated oocytes not presenting relevance. Benzenedicarboxylic acid was present in 13 of the 31 women with 10 or fewer aspirated oocytes and in 16 of the 23 women with more than 10, while cyclohexadecane was observed in 3 of the 23 high-responders, representing 5.56% of all samples and 13.04% of that subgroup. A higher frequency of Benzenedicarboxylic acid, a known phthalate derivative, was observed in women with greater oocyte yield (>10), potentially reflecting metabolic or environmental influences linked to ovarian response and FF composition. Table 8 details the occurrence and relative frequency of VOCs regarding oocyte yield (≤10 vs. >10 aspirated oocytes).

### 3.7. Embryo Quality

Regarding total embryo quality, only the embryos reflecting bad overall developmental potential presented significant relevance towards three compounds. Benzene, tetradecanoic acid trimethylsilylester, and cholesterol, were each present in 2 of the 23 women with poor embryo quality. It should be noted that only 52 samples were studied for the variable embryo quality, not 54. A higher occurrence of benzene- and lipid-related metabolites in the poor-quality embryo group may suggest an association between altered follicular microenvironment composition and impaired embryo development. Table 9 details the occurrence and relative frequency of VOCs detected in the FF of women with bad embryo quality.

### 3.8. β-hCG Outcome

Pregnancy outcomes showed that 2-propenenitrile-3,3-diphenyl and decane were both found in 2 of the 11 women with positive β-hCG, representing 18.18% of this group and 3.07% of all cases, while cyclohexadecane was found in 3 of the 17 women in whom β-hCG was not transferred, corresponding to 17.65% of that subgroup and 5.56% of all cases. A higher detection of aromatic and aliphatic hydrocarbons in women with negative β-hCG outcomes may indicate possible associations between environmental exposure and reduced implantation potential. Positive β-hCG serum levels, regarding successful implantation and early pregnancy, were not favorably correlated with any analytes. Table 10 details the occurrence and relative frequency of VOCs, stratified by β-hCG serum levels (negative: no implantation; no transfer: no embryo was transferred).

### 3.9. Nationality

Geographic variation was also observed, although not across all countries. No significant associations were found between the detected analytes and the FF from Uruguayan or Belgian participants. Phenol and isopropyl myristate were detected in 3 of the 43 Portuguese women, as well as in one Brazilian and one Dutch sample. Palmitic acid methyl ester was identified in five Portuguese samples and in one Brazilian and one Dutch sample, while myristic acid was found in three Portuguese samples and in one Brazilian sample. The trimethylsilyl ester of hexadecanoic acid was detected in five Portuguese, three of the five French, and the Brazilian samples. In contrast, 1-dodecanol, dihydro-methyl-jasmonate, octane-1,1′-oxybis, ethylhexyl-salicylate, nonadecane, propenoic acid 3-(4-methoxyphenyl)-2-ethylhexyl ester, and the trimethylsilyl ester of tetradecanoic acid were each detected in one Portuguese and one Brazilian sample. Oleamide was identified in two Portuguese and one Brazilian sample, ethanol-2-dodecyloxy in one Portuguese and one German sample, and both decane and dodecane-4,6-dimethyl in one Portuguese and one Dutch sample. Variations in compound detection among countries may reflect differences in environmental exposures, dietary habits, or metabolic factors influencing FF composition. Table 11 summarizes the occurrence and relative frequency of VOCs according to the women’s country of birth (Portugal, France, Germany, Brazil, and the Netherlands).

### 3.10. Pregnancy History

Lastly, reproductive history revealed that palmitic acid methyl ester was present in 4 of the 39 women without previous pregnancies, 1 of the 12 with one pregnancy, 1 of the 2 with two pregnancies, and the only woman with three pregnancies. Diisooctylphthalate was detected in three nulliparous women, two of those with one pregnancy, and the woman with three pregnancies. Benzene-2-ethyl-1,4-dimethyl appeared in one nulliparous woman and in one of the two women with two pregnancies. Cyclohexadecane was identified in two nulliparous women and in the woman with three pregnancies, while cyclotetradecane was found in two nulliparous, two uniparous, and the woman with three pregnancies. Similarly, octadecanoic acid methyl ester was detected in two nulliparous, one uniparous, and the woman with three pregnancies, and octadecanoic acid butyl ester in one nulliparous, two uniparous, and the woman with three pregnancies. Undecane-4,8-dimethyl, decane-2,4,6-trimethyl, eicosane, and decane were each found in one nulliparous woman and one of the two women with two pregnancies. Lastly, dodecane-4,6-dimethyl was detected in one nulliparous woman, one of the two women with two pregnancies, and the woman with three pregnancies. Variation in compound detection by parity may reflect cumulative environmental exposures, metabolic adaptations, or reproductive history influencing FF composition. Table 12 details the occurrence and relative frequency of VOCs categorized by their relationship with the number of previous pregnancies.

**Figure 1:**
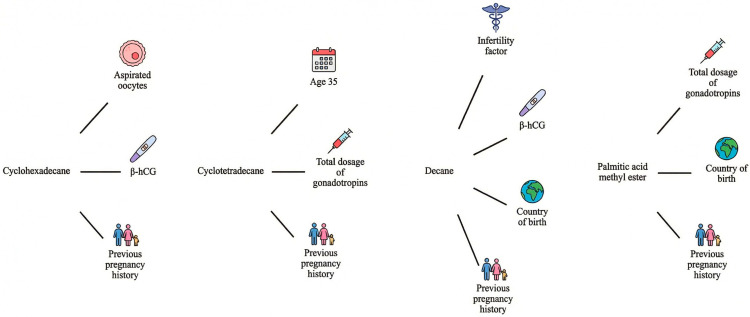
Summary of the most relevant VOCs identified and their associations with the FFRP. The figure integrates the principal compound–parameter relationships observed, providing a visual synthesis of the key findings.

Figure 1 provides an integrative overview of the VOCs identified as the most relevant in this study and their associations with the reproductive parameters evaluated. This summary visualization highlights the compounds that showed the most consistent relationships across the dataset, offering a concise representation of the key findings and facilitating a clearer interpretation of the patterns described in the text. By consolidating the principal VOC–parameter associations, the figure serves as a visual synthesis of the main results and supports the identification of candidate compounds for future targeted investigation.

## 4. Discussion

Across all analyzed FF samples, a wide range of volatile and semi-volatile organic compounds were detected. These compounds varied in frequency across clinical subgroups, suggesting possible interactions between metabolic state, environmental exposure, and ovarian function. Their detection reflects both endogenous lipid metabolism and exogenous contamination pathways that may influence assisted reproductive outcomes.

These findings collectively underscore the complexity of the follicular biochemical environment, where metabolic, endocrine, and environmental inputs converge. Integrating volatilomic profiles with classical metabolomic or proteomic datasets may further enhance understanding of oocyte competence and embryo developmental potential.

### 4.1. Cyclic Hydrocarbons

Several long-chain cyclic hydrocarbons were identified across reproductive and metabolic contexts. Cyclohexadecane appeared in 13% of women with more than ten aspirated oocytes, as well as in women without previous pregnancies and one with three prior pregnancies. This compound’s distribution may indicate lipid accumulation associated with follicular growth rather than infertility per se. Cyclotetradecane was found in 26.3% of women who received gonadotropin doses ≥3000 IU and among those aged 35–39 years or with varying parity histories. Its recurrence in metabolically and hormonally distinct subgroups suggests that cyclic hydrocarbon accumulation may accompany both age- and stimulation-related metabolic changes.

Cyclopentasiloxane-decamethyl was detected in 13.3% of control women, suggesting that siloxane exposure is widespread but may not directly associate with infertility, instead reflecting environmental or cosmetic product exposure.

### 4.2. Aromatic Hydrocarbons

Aromatic hydrocarbons were occasionally detected and appeared to cluster in association with metabolic and embryo quality parameters. Benzene was present in 2 of the 17 samples associated with poor embryo quality, supporting the notion that exposure to aromatic compounds may impair embryonic development through oxidative or genotoxic mechanisms. Benzene is a widespread environmental contaminant, commonly associated with petroleum products, industrial emissions [16], and cigarette smoke [17]. Its adverse effects on human health have been extensively described, particularly its toxic, genotoxic, and carcinogenic properties. Of special concern is its ability to disrupt reproductive processes at multiple regulatory levels, impairing ovarian function, hormone regulation, and fertility in both animals and humans. Occupational and environmental studies report menstrual irregularities [4,17], oligo-menorrhea [4], and reductions in estradiol during the follicular and luteal phases [4,16,17,18]. Associations have also been observed between benzene-related VOCs and altered sex hormone levels, including elevated testosterone in women [16,19]. These findings suggest that benzene disrupts endocrine physiology and ovarian regulatory mechanisms. Regarding assisted reproduction, women with higher intra-follicular benzene levels showed elevated baseline FSH, lower estradiol peaks, fewer oocytes retrieved, and fewer embryos transferred. Importantly, gonadotropin dose and stimulation duration were similar between groups, suggesting that benzene exposure has been associated with reduced ovarian sensitivity to gonadotropins through local follicular toxicity or impaired FSH receptor signaling rather than classical poor ovarian reserve [17]. Smoking was also identified as a major source of intra-follicular benzene [17,20]. Women exposed to cigarette smoke or secondhand smoke exhibited higher FF benzene levels, higher basal FSH, and reduced ovarian response during IVF. Cigarette smoking has been linked to accelerated follicular depletion, earlier menopause, and alterations in ovarian molecular pathways. The dysregulation of over 150 miRNAs has also been linked to smoke-induced ovarian dysfunction and is associated with ovarian carcinogenesis [20].

Benzene-2-ethyl-1,4-dimethyl was identified in 3.7% of all samples, with 15.4% of these cases corresponding to women with a BMI above 25. Given the structural similarities between benzene-2-ethyl-1,4-dimethyl and other benzene derivatives, it is plausible that this compound may share similar health risks, including neurotoxicity and potential reproductive and developmental effects.

Tetramethylbenzene appeared, in total, in 7.4% of all samples, and 75% of the women presenting this compound also exhibited a BMI above 25. This distribution suggests a potential relationship between higher adiposity and the accumulation of lipophilic aromatic compounds, which may exacerbate oxidative stress (OS) in the ovarian microenvironment.

Benzenedicarboxylic acid, a phthalate metabolite also designated o-benzenedicarboxylic acid but mostly as 1,2-Benzenedicarboxylic acid, was notably prevalent, found in 13 of 31 women with ≤10 aspirated oocytes and 16 of 23 women with >10 oocytes, representing one of the most common compounds detected. This high detection rate highlights the ubiquity of phthalate exposure and its infiltration into the ovarian environment, reinforcing the need to consider environmental toxicants as potential modifiers of ART outcomes. Phthalates are a broad family of synthetic compounds structurally derived from 1,2-benzenedicarboxylic acid [21,22]. They exist mainly as esters, but since they are not chemically bound to the polymer matrix, phthalates can leach into the environment and enter the human body through ingestion, inhalation, and dermal absorption [21]. These compounds are widely used as plasticizers to increase flexibility in plastics [21,22], being present in food packaging [21,23], textiles [21], toys, cosmetics [23,24], medical devices [24], and kitchen utensils [23], making exposure nearly unavoidable. Phthalates have been detected in serum, urine [25], amniotic fluid, and ovarian FF, confirming exposure during critical stages of development, including pregnancy [21,22,24]. Phthalates also act through interference with hormone synthesis and signaling [21,23]. They can disrupt ovarian steroidogenesis, alter estradiol and testosterone levels, and impair gonadotropin responses, ultimately reducing oocyte quality, ovulation efficiency, and embryo implantation. Evidence also suggests phthalates may influence endometrial enzymatic activity, potentially contributing to conditions like endometriosis [23]. Their persistence in biological compartments suggests chronic, lifelong exposure. Due to their toxicity, several phthalates, including di(2-ethylhexyl) phthalate (DEHP), dibutyl phthalate (DBP), diisobutyl phthalate (DiBP), benzyl butyl phthalate (BBP), diisopentyl phthalate (DIPP), dimethyl ester of phthalic acid (DMEP), dipropyl phthalate (DPP), di-n-hexyl phthalate (DnHP), and 1,2-benzenedicarboxylic acid, have been restricted worldwide [21]. However, industrial use remains high, particularly in clothing, accessories, and textiles, with phthalate production estimated at nearly 5 billion kilograms annually [21,22,24]. Metabolomic and proteomic analyses confirm that 1,2-benzenedicarboxylic acid is associated with changes in metabolic pathways relevant to reproductive health. It appears in biological networks related to hormone signaling, lipid metabolism, and cellular stress responses, further underscoring its systemic impact. 1,2-benzenedicarboxylic acid and its esters, especially DEP, represent significant environmental and reproductive toxicants. They are linked to infertility risk, altered hormonal regulation, and possible roles in conditions such as endometriosis [21,23].

Diethyl phthalate (DEP) is one of the most common phthalates and a documented endocrine-disrupting chemical (EDC). Epidemiological evidence shows that DEP exposure correlates with reproductive dysfunction, particularly in women with infertility. Associations have been reported between urinary DEP metabolites and increased risk of infertility in women over 35. In younger women, the associations appear weaker, suggesting that age amplifies vulnerability to DEP’s endocrine-disrupting effects [26]. Moreover, BMI modifies the relationship between DEP and infertility. Women with higher BMI values (≥24) exposed to DEP exhibited stronger associations with infertility than leaner women, suggesting that adipose tissue may influence phthalate metabolism or storage, thereby altering endocrine disruption outcomes [26].

Several additional hydrocarbons, including dodecane, dodecane-4,6-dimethyl, and eicosane, were sporadically detected in women from multiple cohorts, including Portuguese and Dutch participants. Their low frequency and broad distribution suggest background exposure or minor metabolic activity rather than direct reproductive significance. Recent studies on VOCs in biological samples have associated dodecane-4-6-dimethyl and 1-dodecanol with sexually transmitted infections. 4,6-dimethyldodecane, also called dodecane-4-6-dimethyl, was present in the urine of patients with *M. genitalium*. Alkanes synthesis is not frequent in bacteria, this being a pathway identified for the first time in cyanobacteria [27]. 1-dodecanol is also a volatile biomarker/fingerprint for the identification of sexually transmitted infections in vaginal discharge and urine [27,28].

### 4.3. Other Endocrine Disruptors and Environmental Contaminants

EDCs are a diverse group of substances that interfere with hormonal systems, causing adverse effects on development, reproduction, metabolism, and general health. EDCs act by targeting nuclear receptors (e.g., estrogen, androgen, pregnane X receptors), modifying hormone synthesis enzymes, and disrupting receptor-mediated signaling [25]. Their effects are variable, depending on the compound, dose, timing of exposure, and individual susceptibility. EDCs have been detected in urine, serum, breast milk, amniotic fluid, and FF, demonstrating lifelong and transgenerational exposure potential [21]. The most concerning effects of EDCs involve the reproductive system. In women, EDC exposure is associated with menstrual irregularities and altered gonadotropin levels; reduced ovarian reserve and impaired response to gonadotropins in IVF; increased risk of infertility [29,30], especially with age and high BMI [30]; PCOS [31,32,33] and endometriosis, possibly mediated by altered steroidogenesis and endometrial enzyme activity; and earlier menopause due to accelerated follicular depletion [30]. Beyond reproduction, EDCs are implicated in thyroid dysfunction, metabolic disorders, obesity, and hormone-related cancers. The impact of EDCs is not uniform across populations. Growing evidence of harm has led to international regulatory actions: the European Union (EU) has restricted several phthalates in consumer products, the US FDA (United States Food and Drug Administration) has reduced approval for phthalates in food contact materials, and per- and polyfluoroalkyl substances (PFAS) regulations are emerging, though global restrictions remain limited [21].

Phenol was detected in 1 of 35 women who received <3000 IU of gonadotropins and 4 of 19 who received ≥3000 IU, representing 9.3% of all samples. It was also present in Portuguese, Brazilian, and Dutch cohorts. Given phenol’s well-established endocrine-disrupting potential, acting through estrogenic, antiestrogenic, and thyroid-modulating pathways, its increased presence in women, requiring higher stimulation, may reflect endocrine disruption that necessitates greater hormonal dosing for follicular recruitment. Phenols are widespread environmental chemicals with known endocrine-disrupting potential, commonly found in consumer products such as plastics, detergents, and cosmetics. Experimental evidence from both in vitro and in vivo studies indicates that phenols can interfere with reproductive function through estrogenic, antiestrogenic, antiandrogenic, and thyroid-disrupting mechanisms. These hormonal interferences may impair fecundability and increase the risk of early pregnancy loss. However, epidemiological studies in non-fertility-treatment populations remain limited and inconsistent. Given these potential risks, reducing preconception exposure to phenol-containing products could provide meaningful public health benefits for reproductive health [34].

Diisooctylphthalate, detected in 3 of 39 women with no previous pregnancies, 2 of 12 women with one, and one woman with three pregnancies, suggests widespread exposure to phthalates. Phthalate esters are known to interfere with estrogen and androgen signaling, and their follicular detection aligns with environmental endocrine disruption, potentially affecting reproductive performance.

### 4.4. Lipid-Derived and Steroidal Compounds

Cholesterol was detected in 2 of the 23 samples from women with poor embryo quality. Although cholesterol is essential for steroidogenesis, excessive levels within the follicular environment can alter local hormone ratios, contributing to oxidative imbalance and reduced oocyte competence. Dyslipidemia, defined by abnormal lipid levels such as high total cholesterol, low-density lipoprotein (LDL), triglycerides, or low high-density lipoprotein (HDL), has been associated with reduced fertility. A prospective cohort study of over 500 couples showed that higher serum free-cholesterol levels were found in both men and women who failed to conceive within 12 months, suggesting a negative impact on natural fertility and time to pregnancy. In reproductive health, cholesterol is indispensable as the precursor of estrogen and progesterone, which coordinate endometrial changes necessary for implantation. Variations in cholesterol profiles across the menstrual cycle reflect increased steroidogenic demand during the implantation window. However, excessive free cholesterol may interfere with reproductive physiology, as seen in infertility associations and findings such as cholesterol-mediated repression of prostaglandin E2 release in renal pathways. Free cholesterol is a double-edged regulator: it is essential for steroidogenesis, immune signaling, and reproduction, but harmful when dysregulated. Elevated serum-free cholesterol is linked to infertility, immune dysfunction, and metabolic disease, underscoring its role as both a biomarker and a potential therapeutic target in reproductive and systemic health [35].

### 4.5. Volatile Lipid Derivatives and Esters

Dihydro-methyl-jasmonate was found in 3.7% of all samples, particularly in women who received ≥3000 IU of gonadotropins and across multiple national groups. This compound, previously proposed as a volatile biomarker in infection and inflammation models, may reflect the activation of oxidative or lipid-signaling cascades within stimulated follicles. Its presence supports its potential as a biomarker of OS and ovarian response to stimulation. Also described as dihydro-methyl-jasmonate, methyl dihydrojasmonate relates to sexually transmitted infections, being consistently detected in both vaginal discharge and urine from patients with *Trichomonas vaginalis* infection. Although its diagnostic strength was lower than that of other VOCs, its presence across different biological fluids suggests that it may serve as a reliable indicator of infection [28]. Chemical analyses of disposable e-cigarettes have identified methyl dihydrojasmonate as one of the flavoring agents present in these products. While its role is primarily to enhance aroma and taste, its inclusion places it within a broader mixture of VOCs, humectants, nicotine, carbonyls, metals, and other additives. Although the specific health effects of methyl dihydrojasmonate from inhalation remain unclear, its presence in disposable e-cigarettes contributes to overall exposure to complex chemical mixtures that have been associated with nicotine dependence, respiratory irritation, and potential carcinogenic risks [36].

Isopropyl myristate, identified in 9.3% of all samples and across several nationalities, was more frequent in women, requiring higher gonadotropin doses. While likely exogenous, its accumulation in FF indicates permeability to environmental lipids and raises questions about interference with follicular steroidogenesis.

Oleamide, present in 5.6% of all samples and in 15.8% of women receiving higher gonadotropin doses, is an endogenous fatty acid amide linked to the endocannabinoid system. Its increased detection may reflect adaptive mechanisms modulating follicular oxidative and inflammatory balance during stimulation.

### 4.6. Straight-Chain and Branched Alkanes

Decane was identified in 3.7% of all samples, occurring in control women and those with positive β-hCG outcomes, indicating possible background lipid oxidation or environmental exposure. Its branched analog, decane-2,4,6-trimethyl, was found in women aged 37–39 years and among those without or with two previous pregnancies, reinforcing the view that these hydrocarbons likely represent metabolic byproducts rather than direct infertility markers.

Undecane, 4,8-dimethyl and pentadecane, each detected in approximately 3.7–5.6% of samples, appeared predominantly in women receiving higher gonadotropin doses (≥3000 IU), suggesting a possible link between ovarian stimulation and the mobilization or accumulation of long-chain hydrocarbons.

Nonadecane appeared in a similar frequency (3.7% of all samples), again primarily in women receiving higher gonadotropin doses or from specific geographic backgrounds, emphasizing the influence of both environmental and procedural factors.

### 4.7. Fatty Acids and Their Derivatives

Fatty acid derivatives were among the most frequently detected compounds. Hexadecanoic acid trimethylsilyl ester was found in 16.7% of all samples, corresponding to 23.1% of women who were the primary cause of infertility. Palmitic acid methyl ester (detected in 5 of 19 women receiving ≥3000 IU; 9.3% overall) was observed primarily in Portuguese samples and also in single samples from Brazil and the Netherlands. By contrast, the trimethylsilyl ester of hexadecanoic acid was detected in five Portuguese samples and in three of five French samples, indicating a distinct geographical pattern for this fatty acid derivative. These fatty acid esters likely reflect altered lipid metabolism under hormonal stimulation, consistent with previous reports linking palmitate accumulation to OS and impaired oocyte maturation.

Palmitic acid is one of the most abundant saturated fatty acids (SFAs) in human plasma and FF, commonly found alongside stearic and oleic acids. In physiological conditions, palmitic acid serves as a key energy substrate and a component of membrane lipids. However, excessive accumulation is often linked to metabolic disorders such as obesity, insulin resistance, and PCOS, and can exert deleterious effects on ovarian and reproductive function.

In women with PCOS, plasma and FF concentrations of palmitic acid are significantly elevated, reflecting broader disturbances in lipid metabolism [37]. Studies show that this increase occurs even in non–insulin-resistant phenotypes, suggesting that an abnormal fatty acid metabolism itself plays a pathogenic role independent of obesity or insulin resistance. This indicates the profound disruption of fatty acid oxidation and energy regulation in affected individuals [37,38,39].

Within the ovarian microenvironment, palmitic acid is the predominant fatty acid in FF (often exceeding 50% of total fatty acids), and its accumulation correlates negatively with oocyte maturation, embryo quality [40,41,42], and fertilization rates [40]. Mechanistically, palmitic acid has been shown to induce apoptosis [40,41,42] and endoplasmic reticulum (ER) stress in granulosa and cumulus cells [40], which may contribute to impaired folliculogenesis and reduced developmental competence of oocytes. This cytotoxic effect is dose-dependent, with concentrations above 100 µM significantly reducing GC viability through increased caspase-3 and CHOP expression, activation of pro-apoptotic BAX, inhibition of Akt phosphorylation, and accumulation of ceramides and reactive oxygen species (ROS). These stress responses are thought to contribute to mitochondrial dysfunction, oxidative damage, and DNA fragmentation within ovarian cells [41].

Experimental studies further demonstrate that palmitic acid exposure enhances ER stress markers (GRP78, CHOP) and triggers apoptosis via activation of JNK signaling, which can be reversed by JNK inhibition. In oocytes and embryos, excessive palmitic acid reduces cell proliferation, alters glucose metabolism, and increases apoptosis in trophoblast and blastocyst cells, compromising embryonic viability. Moreover, palmitic acid-treated GCs exhibit impaired insulin and IGF-1 signaling, contributing to local insulin resistance, a hallmark of PCOS pathophysiology [40].

Interestingly, the biological impact of palmitic acid may vary depending on hormonal context. Under conditions of high estradiol levels, palmitic acid can modestly enhance FSH and LH receptor expression or promote estrogen synthesis, suggesting a potential adaptive or compensatory role in certain endocrine environments. Nonetheless, the prevailing evidence indicates that sustained elevations in palmitic acid are detrimental to oocyte quality, GC, survival, and embryo development [41].

Beyond PCOS, increased palmitic acid has also been associated with DOR and poorer IVF outcomes. Metabolomic analyses reveal that palmitic acid, together with stearic acid and specific phosphatidylcholine derivatives, can discriminate between women with DOR and those with normal ovarian reserve, emphasizing its relevance as a potential biomarker of reproductive competence [40].

Octadecanoic acid and its methyl and butyl esters were found in 7.4–9.3% of samples, primarily among women receiving higher gonadotropin doses or with varying parity. Their detection points to increased lipid turnover or metabolic stress within the follicular environment, processes that may influence membrane integrity and hormone signaling.

Octadecanoic acid, commonly known as stearic acid, is an 18-carbon SFA naturally found in the body and within the FF surrounding the developing oocyte. It represents one of the most abundant fatty acids in this environment, typically accompanied by palmitic acid and oleic acid [41]. Together, these fatty acids form a major portion of the follicular lipid composition and play crucial roles in oocyte maturation, fertilization, and early embryo development [40,41,42,43].

Stearic acid is primarily derived from dietary fats and local lipid metabolism [42]. In women undergoing IVF or intracytoplasmic sperm injection (ICSI), studies have shown that stearic acid can account for approximately 26% of the total FF fatty acid content [40]. While fatty acids are essential for energy production and membrane synthesis, their balance and concentration are critical: both deficiencies and excesses can disrupt reproductive function. Excess accumulation of SFA (including stearic acid) has been associated with increased OS, lipid peroxidation, and an altered metabolic profile. These factors are known contributors to reduced oocyte quality and fertility potential [41,43].

Multiple studies have demonstrated that elevated levels of stearic acid in FF are negatively correlated with oocyte maturation rate (Metaphase II oocytes), embryo quality [40,41,42], and fertilization rate [38,40,43]. Elevated stearic acid levels have been associated with GC apoptosis [40,41,42], mitochondrial dysfunction [41], and ER stress [40], all of which impair the oocyte’s ability to mature and fertilize successfully [40,41,42,43]. These effects are often dose-dependent, and even moderate elevations (>100 μM) can reduce GC viability and trigger apoptotic pathways involving caspase-3 activation, CHOP, and BAX expression [41]. Mechanistically, stearic acid has been shown to induce ROS formation through β-oxidation overload, leading to OS that damages lipids, proteins, and DNA [43]. This cytotoxic stress disrupts cumulus cell expansion, reduces oocyte competence, and ultimately compromises blastocyst formation. Embryos derived from environments rich in stearic and palmitic acids show higher apoptotic activity, altered metabolism, and diminished developmental potential [40,41,42].

Stearic acid also affects ovarian metabolic signaling by disrupting insulin and IGF-1 pathways, reducing glucose uptake and promoting insulin resistance, a hallmark of PCOS. Elevated stearic acid in the serum and FF of PCOS patients reflects dyslipidemia and androgen excess, contributing to OS and impaired folliculogenesis. These alterations appear even in non-obese PCOS cases, suggesting that abnormal fatty acid metabolism itself plays a pathogenic role. While generally linked to adverse outcomes, stearic acid may under certain hormonal conditions—such as high estradiol levels—support estrogen synthesis or FSH/LH receptor expression in GCs, indicating a context-dependent effect [41].

Beyond PCOS, altered stearic acid metabolism has been observed in other reproductive disorders such as endometriosis and DOR. In DOR patients, distinct metabolic signatures, including elevated stearic acid, have been linked to poor IVF outcomes [44]. Similarly, studies integrating metabolomic profiling reveal that stearic acid, along with palmitic and linoleic acids, is positively associated with PCOS prevalence and reduced oocyte maturity, independent of obesity. These findings suggest that stearic acid serves as both a metabolic indicator and a potential effector of reproductive dysfunction. Its accumulation may reflect underlying OS, hyperandrogenism, or dietary lipid imbalance, all factors capable of compromising ovarian performance [39,40,41].

### 4.8. Aldehydes and Related Compounds

Hexadecanal, a long-chain aldehyde derived from sphingolipid metabolism, was identified in 6 of 15 control women but only 3 of 39 infertile women, suggesting a relative reduction in this metabolite among infertility cases. This imbalance may indicate impaired aldehyde detoxification or altered sphingolipid metabolism, both of which are associated with oxidative damage and suboptimal follicular conditions. Hexadecanal is a type of long-chain fatty aldehyde, a molecule that forms naturally in the body during the breakdown of certain fats called sphingolipids. While sphingolipids are essential components of cell membranes, their breakdown can release reactive aldehydes like hexadecanal, which are toxic when they accumulate. These aldehydes can damage cells by binding to proteins, lipids, or DNA, leading to inflammation, OS, and cellular dysfunction. Proper lipid and aldehyde balance is crucial for hormone production, oocyte maturation, and implantation. Excessive aldehyde accumulation, including compounds like hexadecanal, can damage ovarian or endometrial cells, disrupt hormonal signaling, and increase OS, all of which have been linked to infertility and disorders such as PCOS [45].

Oxirane-hexadecyl, detected in eight samples (five controls and three infertile women), followed a similar trend, reinforcing the potential metabolic divergence between fertile and infertile individuals.

### 4.9. Silicon-Based Compounds

Tetradecamethylcycloheptasiloxane and eicosamethylcyclodecasiloxane were detected in 64.8% and 5.6% of all samples, respectively, with the majority (85.7%) occurring in women with BMI ≤ 25. Their prevalence, especially in women of normal weight, suggests consistent environmental exposure, likely from cosmetics or personal care products, rather than a pathophysiological origin. Nevertheless, given their lipophilic character, these compounds could accumulate in the FF, potentially influencing membrane dynamics and redox balance.

Taken together, these patterns suggest that specific VOC classes, particularly phthalates, phenols, and fatty acid derivatives, may act as candidate biomarkers of oxidative stress and impaired follicular homeostasis. The validation of these findings in larger, independent ART cohorts and through longitudinal sampling could clarify the predictive potential of FF volatilomics in clinical practice.

## 5. Conclusions

The compounds detected in each sample were correlated with several fertilization-related parameters. After FDR correction, only the correlation with the lowest *p*-value remained statistically significant (q = 0.044), while all others did not reach significance (q > 0.05). Thus, although some associations appeared in uncorrected analyses, most compound–clinical parameter relationships were weak or limited by the sparse distribution of VOCs and the sample size. Chi-square tests provided an exploratory view of presence/absence patterns, even though few reached statistical significance. Several VOCs, particularly cyclohexadecane, cyclotetradecane, decane, and palmitic acid methyl ester, showed correlations with multiple FFRP, indicating their relevance for further study. Overall, the identified VOCs may reflect dysregulated biochemical pathways and could serve as biomarkers for reproductive disturbances that compromise ART success.

The volatilomic profiling approach used here demonstrates the feasibility of exploring VOC-based biomarkers linked to fertilization, infertility, and reproductive outcomes. Three major themes emerged: (1) environmental exposure (siloxanes, phthalates, phenolics), (2) metabolic lipid remodeling (aldehydes, fatty acids, esters), and (3) endocrine modulation (steroidal and amide derivatives). This integrative perspective highlights how environmental and metabolic factors converge to influence reproductive physiology.

Despite promising findings, the study has limitations: a relatively small sample size, the cross-sectional design, potential environmental contamination, and the semi-quantitative nature of the analysis. Limited biological knowledge for some compounds also constrains mechanistic interpretation. Moreover, although different infertility-related clinical characteristics may act as relevant confounding factors, these could not be integrated into the present analysis due to sample size limitations and the high dimensionality of the volatilomic data. Nevertheless, we fully agree that incorporating detailed clinical characteristics as additional variables represents an important next step, and this will be addressed in future studies using larger, more balanced cohorts. Future work should include quantitative, targeted analyses of candidate VOCs and validation in larger, independent, and ideally longitudinal cohorts. Integrating volatilomics with metabolomics and proteomics will provide a more holistic view of the follicular environment and its role in disorders such as PCOS. Increasing sample diversity and expanding the literature on VOCs will enhance the robustness and biological interpretability of these findings.

In summary, this study lays a foundation for future mechanistic research and highlights the potential of VOCs as biomarkers in personalized reproductive medicine, while emphasizing cautious interpretation due to data sparsity, small sample size, and multiple-testing constraints.

## Figures and Tables

**Table 1:** Female fertilization-related parameters (FFRPs).

FFRP and Categories	Min–Max	Median (Mean ± SD)	*n* (%)
**Age (years)**	**19–39**	**35 (34.46 ± 3.68)**	-
<30	-	-	4 (7.4)
≥30	-	-	50 (92.6)
<35	-	-	25 (46.30)
≥35	-	-	29 (53.70)
<37	-	-	35 (64.81)
≥37	-	-	19 (35.19)
**BMI (kg/m^2^)**	**17.0–32.0**	**23.0 (23.41 ± 3.61)**	-
≤25	-	-	41 (75.93)
>25	-	-	13 (24.07)
**Smoking habits**	-	-	-
Never	-	-	43 (79.63)
Previous	-	-	6 (11.11)
Present	-	-	5 (9.26)
**Infertility factor**	-	-	-
Yes	-	-	39 (72.22)
No	-	-	15 (27.78)
**Time trying to conceive (months)**	**18–192**	**38.5 (52.74 ± 35.85)**	-
≤24	-	-	2 (3.70)
25–48	-	-	30 (55.56)
≥48	-	-	22 (40.74)
**Time trying to conceive—dichotomized**	-	-	-
≤48	-	-	33 (61.11)
>48	-	-	21 (38.89)
**Total dosage of gonadotropins (IU)**	**150–3900**	**2250 (2383.62 ± 783.73)**	-
<3000	-	-	35 (64.81)
≥3000	-	-	19 (35.19)
**Aspirated oocytes**	**0–23**	**9 (9.87 ± 5.39)**	-
≤10	-	-	31 (57.41)
>10	-	-	23 (42.59)
≤6	-	-	10 (18.52)
>6	-	-	44 (81.48)
**Transferred embryo quality**	-	-	-
Excellent	-	-	14 (25.93)
Good	-	-	16 (29.63)
Medium	-	-	8 (14.81)
Bad	-	-	0 (0)
No transfer	-	-	16 (29.63)
**Total embryo quality**	-	-	-
Good	-	-	35 (67.31)
Bad	-	-	17 (32.69)
**β-hCG outcome**	-	-	-
Positive	-	-	11 (20.37)
Negative	-	-	26 (48.15)
No transfer	-	-	17 (31.48)
**Country of birth**	-	-	-
Portugal	-	-	43 (79.63)
France	-	-	5 (9.26)
Germany	-	-	2 (3.70)
Brazil	-	-	1 (1.85)
Uruguay	-	-	1 (1.85)
Netherlands	-	-	1 (1.85)
Belgium	-	-	1 (1.85)
**Number of previous pregnancies**	-	-	
0	-	-	39 (72.22)
1	-	-	12 (22.22)
2	-	-	2 (3.70)
3	-	-	1 (1.85)

Age was analyzed using clinically relevant thresholds (<30/≥30, <35/≥35, and <37/≥37 years), reflecting established cut-offs associated with changes in ovarian reserve and reproductive prognosis. BMI categories were defined as ≤25 kg/m^2^ (normal weight) and >25 kg/m^2^ (overweight). Smoking habits were classified as never, previous, or present smokers. The infertility factor indicates whether the female partner was the primary cause of infertility. Time trying to conceive was evaluated using the categories ≤24 months, 25–48 months, and ≥48 months. Total gonadotropin dosage refers to the cumulative dose administered during ovarian stimulation (<3000 IU or ≥3000 IU). Aspirated oocytes were stratified according to retrieval number (≤10/>10 and ≤6/>6). β-hCG serum levels were grouped as positive (successful implantation), negative (no implantation), or no transfer (no embryo transferred). Country of birth and previous pregnancy history (0, 1, 2, or 3 prior pregnancies) were included as demographic variables. The median, as well as the mean ± standard deviation, was computed using IBM SPSS Statistics (v.29).

**Table 2:** Statistical associations between volatile organic compounds (VOCs) detected in follicular fluid (FF) and clinical, demographic, and reproductive parameters in women undergoing assisted reproductive procedures.

	Benzene	Benzene-2-ethyl-1-4-dimethyl	Benzenedicarboxylic Acid	Cholesterol	Cyclohexadecane	Cyclopentasiloxane-decamethyl	Cyclotetradeca	Decane	Decane-2-4-6-trimethyl	Dihydro-methyl-jasmonate	Diisooctylphthalate	Dodecane-4-6-dimethyl	1-dodecanol	Eicosamethylcyclodecasiloxane	Eicosane	Ethanol-2-dodecyloxy	Ethylhexyl-salicylate	Hexadecanal	Hexadecanoic acid trimethylsilylester	Isopropyl-myristate	Myristic Acid	Nonadecane	Nonadecanone	Octadecan-1-ol trimethylsilyl Ether	Octadecanoic Acid	Octadecanoic Acid Butyl Ester	Octadecanoic Acid Methyl Ester	Octadecyloxy-1-1-2-2 -tetradeuteroethanol	Octane-1-1-oxybis	Oleamide	Oxirane-hexadecyl	Palmitic Acid Methyl Ester	Palmitic Acid	Pentadecane	Phenol	2-propenenitrile-3-3-diphenyl	Propenoic acid 3-4-methoxyphenyl- 2-ethylhexyl-ester	Stearyl Alcohol	Tetradecamethylcycloheptasiloxane	Tetradecane	Tetradecanoic Acid Trimethylsilyl Ester	Tetramethylbenzene	Tetratriacontane	Trimethylsiloxyhexadecane	Undecane-4-8-dimethyl
Age 35							X																																						
Age 37									X																															X					X
BMI		X												X										X															X			X			
SH																							X															X					X		
IF						X		X										X	X												X														
TDG							**X**			X			X				X			X		X			X		X	X	X	X		X	X	X	X		X	X						X	
AO					X																																								
TEQ	X			X																																					X				
β-hCG					X			X																												X									
CB								X		X		X	X			X	X		X	X	X		X						X	X		X			X		X				X				
PPH		X			X		**X**	X	X		X	X			X											X	X					X													X

Chi-square tests (χ^2^) were applied; for variables with low expected frequencies in certain categories (e.g., country of birth), Fisher’s exact test was used to ensure analytical validity (IBM SPSS Statistics (version 29)). The table summarizes compound-specific correlations with age (Age 35; Age 37), body mass index (BMI), smoking habits (SH), infertility factor (IF), total dosage of gonadotropins (TDG), aspirated oocytes g10 (AO), transferred embryo quality (TEQ), β-hCG serum levels (β-hCG), country of birth (CB), and previous pregnancy history (PPH).

**Table 3:** Detection frequency of VOCs in FF according to age groups (≥35; ≥37). Percentage values within the group and regarding the overall samples. Data analyzed using IBM SPSS Statistics (version 29).

Compound	Age					
	≥35 (n = 29)		Overall (54)	≥37 (n = 19)		Overall (54)
Cyclotetradecane	5	17.2%	9.3%	-	-	-
Undecane-4,8-dimethyl	-	-	-	2	10.5%	3.7%
Decane-2,4,6-trimethyl	-	-	-	2	10.5%	3.7%
Tetradecane	-	-	-	2	10.5%	3.7%

**Table 4:** Detection frequency of VOCs in FF according to body mass index (BMI; ≤25 kg/m^2^ (normal weight); >25 kg/m^2^ (overweight)). Percentage values within the group and regarding the overall samples. Data analyzed using IBM SPSS Statistics (version 29).

Compound	BMI					
	≤25 (n = 41)		Overall (54)	>25 (n = 13)		Overall (54)
Tetradecamethylcycloheptasiloxane	30	73.2%	55.6%	5	38.5%	9.3%
Benzene-2-ethyl-1,4-dimethyl	-	-	-	2	15.4%	3.7%
Tetramethylbenzene	1	2.4%	1.9%	3	23.1%	5.6%
Eicosamethylcyclodecasiloxane	2	4.9%	3.7%	3	23.1%	5.6%
Octadecan-1-ol trimethylsilyl ether	2	4.9%	3.7%	3	23.1%	5.6%

**Table 5:** Detection frequency of VOCs in FF according to smoking habits (never—never smoked; previous—smoked but does not anymore; present—smokes actively). Percentage values within the group and regarding the overall samples. Data analyzed using IBM SPSS Statistics (version 29).

Compound	Smoking Habits							
	Never (n = 43)		Overall (54)	Previous (n = 6)		Overall (54)	Active (n = 5)	Overall (54)
2-Nonadecanone	-	-	-	1	16.7%	1.9%	-	-
Tetratriacontane	-	-	-	1	16.7%	1.9%	-	-
Stearyl alcohol	4	9.3%	7.4%	3	50%	5.6%	1	20%

**Table 6:** Detection frequency of VOCs in FF according to infertility factor (yes—is the primordial cause of infertility of the couple; no—is not the primordial cause of infertility of the couple). Percentage values within the group and regarding the overall samples. Data analyzed using IBM SPSS Statistics (version 29).

Compound	Infertility Factor					
	Yes (n = 39)		Overall (54)	No (n = 15)		Overall (54)
Hexadecanal	5	12.8%	9.3%	6	40.0%	11.11%
Oxirane-hexadecyl	3	7.7%	5.6%	5	33.3%	9.3%
Hexadecanoic acid trimethylsilylester	9	23.1%	16.7%	-	-	-
Cyclopentasiloxane-decamethyl	-	-	-	2	13.3%	3.70%
Decane	-	-	-	2	13.3%	3.70%

**Table 7:** Detection frequency of VOCs in FF according to the total dosage of gonadotropins received during ovarian stimulation (women receiving total gonadotropin doses <3000 IU; women receiving doses ≥3000 IU). Percentage values within the group and regarding the overall samples. Data analyzed using IBM SPSS Statistics (version 29).

Compound	Total Dosage of Gonadotropins 3000					
	<3000 (n = 35)		Overall (54)	≥3000 (n = 19)		Overall (54)
Palmitic acid	3	8.6%	5.6%	8	42.1%	14.8%
Phenol	1	2.9%	1.9%	4	21.1%	7.4%
Palmitic acid–methyl ester	2	5.7%	3.7%	5	26.3%	9.3%
Isopropyl myristate	1	2.9%	1.9%	4	21.1%	7.4%
Stearyl alcohol	2	5.7%	3.7%	6	31.6%	11.1%
Octadecanoic acid	-	-	-	5	26.3%	9.3%
Trimethylsiloxyhexadecane	-	-	-	2	10.5%	3.7%
Cyclotetradecane	-	-	-	5	26.3%	9.3%
Octadecanoic acid methyl ester	-	-	-	4	21.1%	7.4%
Pentadecane	-	-	-	2	10.5%	3.7%
Octadecyloxy-1-1-2-2-tetradeuteroethanol				3	15.8%	5.6%
1-Dodecanol	-	-	-	2	10.5%	3.7%
Dihydro-methyl-jasmonate	-	-	-	2	10.5%	3.7%
Octane-1,1-oxybis	-	-	-	2	10.5%	3.7%
Ethylhexyl salicylate	-	-	-	2	10.5%	3.7%
Nonadecane	-	-	-	2	10.5%	3.7%
2-Propenoic acid 3-(4-methoxyphenyl)-2-ethylhexyl ester	-	-	-	2	10.5%	3.7%
Oleamide	-	-	-	3	15.8%	5.6%

**Table 8:** Detection frequency of VOCs in FF according to the number of aspirated oocytes (occurrence regarding g10: ≤10 and >10). Percentage values within the group and regarding the overall samples. Data analyzed using IBM SPSS Statistics (version 29).

Compound	Aspirated Oocytes g10					
	≤10 (n = 31)		Overall (54)	>10 (n = 23)		Overall (54)
Benzenedicarboxylic acid	13	41.9%	24.1%	16	69.6%	29.6%
Cyclohexadecane	-	-	-	3	13.0%	5.6%

**Table 9:** Detection frequency of VOCs in FF according to total embryo quality transferred (bad embryo quality). Percentage values within the group and regarding the overall samples. Data analyzed using IBM SPSS Statistics (version 29).

Compound	Total Embryo Quality		
	Bad Embryo Quality (n = 17)		Overall (54)
Benzene	2	11.8%	3.8%
Tetradecanoic acid trimethylsilyl ester	2	11.8%	3.8%
Cholesterol	2	11.8%	3.8%

**Table 10:** Detection frequency of VOCs in FF according to β-hCG serum levels (negative: no implantation; no transfer: no embryo was transferred). Percentage values within the group and regarding the overall samples. Data analyzed using IBM SPSS Statistics (version 29).

Compound	β-hCG					
	Never (n = 11)		Overall (54)	No Transfer (n = 17)		Overall (54)
2-Propenenitrile-3,3-diphenyl	2	18.2%	3.7%	-	-	-
Cyclohexadecane	-	-	-	3	17.6%	5.6%
Decane	2	18.2%	3.7%	-	-	-

**Table 11:** Detection frequency of VOCs in FF according to participants’ country of birth (Portugal; France; Germany; Brazil; Netherlands). Percentage values within the group and regarding the overall samples. Data analyzed using IBM SPSS Statistics (version 29).

Compound	Country of Birth														
	Portugal (n = 43)		Overall (54)	France (n = 5)		Overall (54)	Germany (n = 2)		Overall (54)	Netherlands (n = 1)		Overall (54)	Brazil (n = 1)		Overall (54)
Phenol	3	7.0%	5.6%	-	-	-	-	-	-	1	100%	1.9%	1	100%	1.9%
Palmitic acid methyl ester	5	11.6%	9.3%	-	-	-	-	-	-	1	100%	1.9%	1	100%	1.9%
Myristic acid	3	7.0%	5.6%	-	-	-	-	-	-	-	-	-	1	100%	1.9%
Isopropyl myristate	3	7.0%	5.6%	-	-	-	-	-	-	1	100%	1.9%	1	100%	1.9%
Hexadecanoic acid trimethylsilylester	5	11.6%	9.3%	3	60.0%	5.6%	-	-	-	-	-	-	1	100%	1.9%
1-dodecanol	1	2.3%	1.9%	-	-	-	-	-	-			-	1	100%	1.9%
Dihydro-methyl-jasmonate	1	2.3%	1.9%	-	-	-	-	-	-	-	-	-	1	100%	1.9%
Octane-1,1-oxybis	1	2.3%	1.9%	-	-	-	-	-	-	-	-	-	1	100%	1.9%
Ethylhexyl salicylate	1	2.3%	1.9%	-	-	-	-	-	-	-	-	-	1	100%	1.9%
Tetradecanoic acid trimethylsilylester	1	2.3%	1.9%	-	-	-	-	-	-	-	-	-	1	100%	1.9%
Nonadecane	1	2.3%	1.9%	-	-	-	-	-	-	-	-	-	1	100%	1.9%
Propenoic acid 3,4-methoxyphenyl-2-ethylhexyl ester	1	2.3%	1.9%	-	-	-	-	-	-	-	-	-	1	100%	-
Oleamide	2	4.7%	3.7%	-	-	-	-	-	-	-	-	-	1	100%	-
Ethanol-2-dodecyloxy	1	2.3%	1.9%	-	-	-	1	50.0%	1.9%	-	-	-	-	-	-
Decane	1	2.3%	1.9%	-	-	-	-	-	-	1	100%	1.9%	-	-	-
Dodecane-4,6-dimethyl	1	2.3%	1.9%	-	-	-	1	50.0%	1.9%	-	-	-	-	-	-

**Table 12:** Detection frequency of VOCs in FF according to the number of previous pregnancies (Nº of previous pregnancies: 0; 1; 2; 3). Percentage values within the group and regarding the overall samples. Data analyzed using IBM SPSS Statistics (version 29).

Compound	Nº of Previous Pregnancies											
	0 (n = 39)		Overall (54)	1 (n = 12)		Overall (54)	2 (n = 2)		Overall (54)	3 (n = 1)		Overall (54)
Palmitic acid methyl ester	4	10.3%	7,4%	1	8.3%	1.9%	1	50.0%	1.9%	1	100.0%	1.9%
Diisooctylphthalate	3	7.7%	5.6%	2	16.7%	3.7%	-	-	-	1	100.0%	1.9%
Benzene-2-ethyl-1,4-dimethyl	1	2.6%	1.9%	-	-	-	1	50.0%	1.9%	-	-	-
Cyclohexadecane	2	5.1%	3.7%	-	-	-	-	-	-	1	100.0%	1.9%
Cyclotetradecane	2	5.1%	3.7%	2	16.7%	3.7%	-	-	-	1	100.0%	1.9%
Octadecanoic acid methyl ester	2	5.1%	3.7%	1	8.3%	1.9%	-	-	-	1	100.0%	1.9%
Octadecanoic acid butyl ester	1	2.6%	1.9%	2	16.7%	3.7%	-	-	-	1	100.0%	1.9%
Undecane-4,8-dimethyl	1	2.6%	1.9%	-	-	-	1	50.0%	1.9%	-	-	-
Decane-2,4,6-trimethyl	1	2.6%	1.9%	-	-	-	1	50.0%	1.9%	-	-	-
Eicosane	1	2.6%	1.9%	-	-	-	1	50.0%	1.9%	-	-	-
Decane	1	2.6%	1.9%	-	-	-	1	50.0%	1.9%	-	-	-
Dodecane-4,6-dimethyl	1	2.3%	1.9%	-	-	-	1	50.0%	1.9%	1	100.0%	1.9%

## Data Availability

Data is contained within the article.

## Abbreviations

The following abbreviations are used in this manuscript:

VOCs	Volatile organic compounds
ART	Assisted reproductive techniques
FF	Follicular fluid
FFRP	Female fertilization-related parameters
GC–MS	Gas chromatography–mass spectrometry
BMI	Body mass index
PCOS	Polycystic ovary syndrome
DOR	Diminished ovarian reserve
GC	Granulosa cell
POF	Premature ovarian failure
IVF	*In vitro fertilization*
PDMS	Polydimethylsiloxane
SPME	Solid-phase microextraction
SH	Smoking habits
IF	Infertility factor
TDG	Total dosage of gonadotropins
AO	Aspirated oocytes g10
TEQ	Transferred embryo quality
CB	Country of birth
PPH	Previous pregnancy history
OS	Oxidative stress
DEHP	Di(2-ethylhexyl) phthalate
DBP	Dibutyl phthalate
DiBP	Diisobutyl phthalate
BBP	Benzyl butyl phthalate
DIPP	Diisopentyl phthalate
DMEP	Dimethyl ester of phthalic acid
DPP	Dipropyl phthalate
DnHP	Di-n-hexyl phthalate
DEP	Diethyl phthalate
EDC	Endocrine-disrupting chemical
EU	European Union
US	United States
FDA	Food and Drug Administration
PFAS	Per- and Polyfluoroalkyl Substances
LDL	Low-density lipoprotein
HDL	High-density lipoprotein
SFA	Saturated fatty acid
ER	Endoplasmic reticulum
ROS	Reactive oxygen species
